# Supplementary material for: Accurate spatiotemporal predictions of daily stream temperature from statistical models accounting for interactions between climate and landscape
Source: PeerJ. 2019 Nov 12;7:e7892. doi: 10.7717/peerj.7892 (PMC6857678; doi:10.7717/peerj.7892)
Supplement: Supplemental Information 1 — This document provides a more in-depth interpretation and visualization of the variable effects and interactions included in the stream temperature models than could be provided within the article. [file peerj-07-7892-s001.pdf]

Supplementary Document

Analysis of interactions between climatic and spatial variables for the manuscript entitled "*Accounting for interactions between climate and landscape in spatiotemporal models to accurately estimate daily stream temperature*"

INTRODUCTION

This supplementary document presents the interpretation of covariate relationships and interactions utilized in statistical stream temperature models from the manuscript entitled "*Accounting for interactions between climate and landscape in spatiotemporal models to accurately estimate daily stream temperature*". Presented surface plots represent model predicted stream temperature with variation in two variables assuming all other variables in the model are held at their median value. The presented plots represent examples of relationships that aligned with hypothesized effects of variables and were retained in model selection. Variables and interactions were categorized as climate-climate interactions, climate-spatial interactions, and spatial-spatial interactions. We identified relationships that were found to be consistent across study watersheds as "universal" and those that were watershed-specific or less consistent in form as "local". While we only utilized predicted relationships that aligned with hypothesized relationships, the exact nature and strength of such relationship depended on the local characteristics of each study watershed.

UNIVERSAL RELATIONSHIPS

Universal relationships were those that were included in most or all models with consistent form.

CLIMATE x CLIMATE VARIABLES AND INTERACTIONS

Climatic effects on steam temperature were largely consistent across watersheds.

***Temperature change* ($T\Delta_a$) and averaged air temperature variables ($T5_a$ or $T3_a$)**

Averaged air temperature variables ($T5_a$ or $T3_a$) were the primary influence on stream temperature in spring and fall period models; *Temperature change* ($T\Delta a$) had a smaller yet significant impact on water temperature (Figure A1). We did not find that an interaction between the two air temperature variables was useful and they are accordingly plotted together solely to demonstrate their relative importance. GAM models captured the commonly observed S shaped relationship between air temperature and stream temperature. At high air temperatures the relationship between stream temperature and air temperature flattens due to evaporative cooling and at low temperatures the relationship flattens as stream temperature approaches freezing. $T5_a$ was the better variable for the spring while $T3_a$ was better in the fall. This is likely due to generally lower discharge in the fall period leaving streams with less thermal inertia allowing for a more rapid influence of air temperature on stream temperature. Note, that depending on the range of stream temperatures experienced in a certain watershed, the relationship with air temperature may only express flattening at one end, or if restricted to middle ranges, remain linear. We generally saw a clearer flattening of the relationship at lower air temperatures in the spring models of the Wenatchee and the Chiwawa, potentially due to these being the most mountainous basins and the spring period including the majority of the coldest winter months.

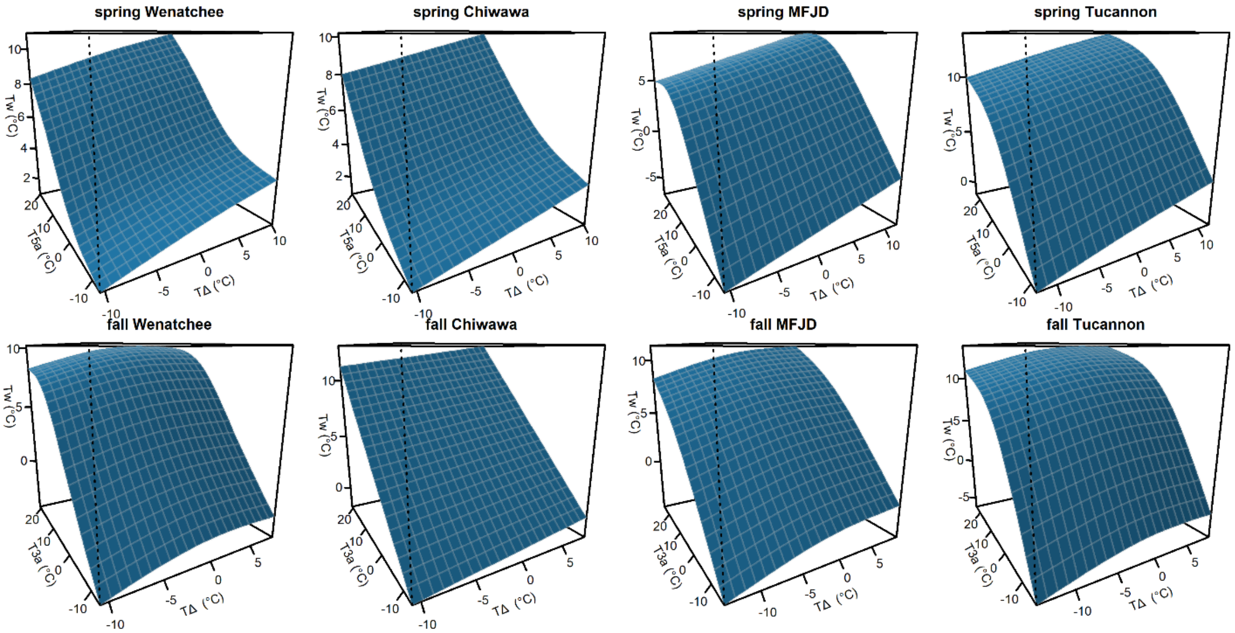

Figure SD1: Conditional model surfaces showing the relationship between averaged air temperature variables ($T5_a$ or $T3_a$) and temperature change ($T\Delta_a$) for the best spring and fall models in all study basins.

Day of year **(*D*) interacting with averaged air temperature variables (*$T5_a$* or *$T3_a$*)**

Including *Day of year* captured predictable seasonal changes in stream temperature while helping to account for seasonal effects distinct from air temperature (e.g. day length, solar radiation due to solar angle). Interactions between air temperature and *Day of year* variables suggest that variability in air temperature had a subdued effect on stream temperature in the winter months compared to the summer months, particularly in the early days of the spring model. The early part of the spring period represents mid-winter and thus air temperatures below or near freezing, where the relationship with stream temperature is flat, were common during this period in all study watersheds. The flexibility of GAM models also allowed them to capture part of the cooling influence of the spring snowpack melt in this interaction, as evidenced by a depression in predicted stream temperatures at high air temperatures in the mountainous Wenatchee and Chiwawa watersheds during the mid-spring, which represents the peak melt period.

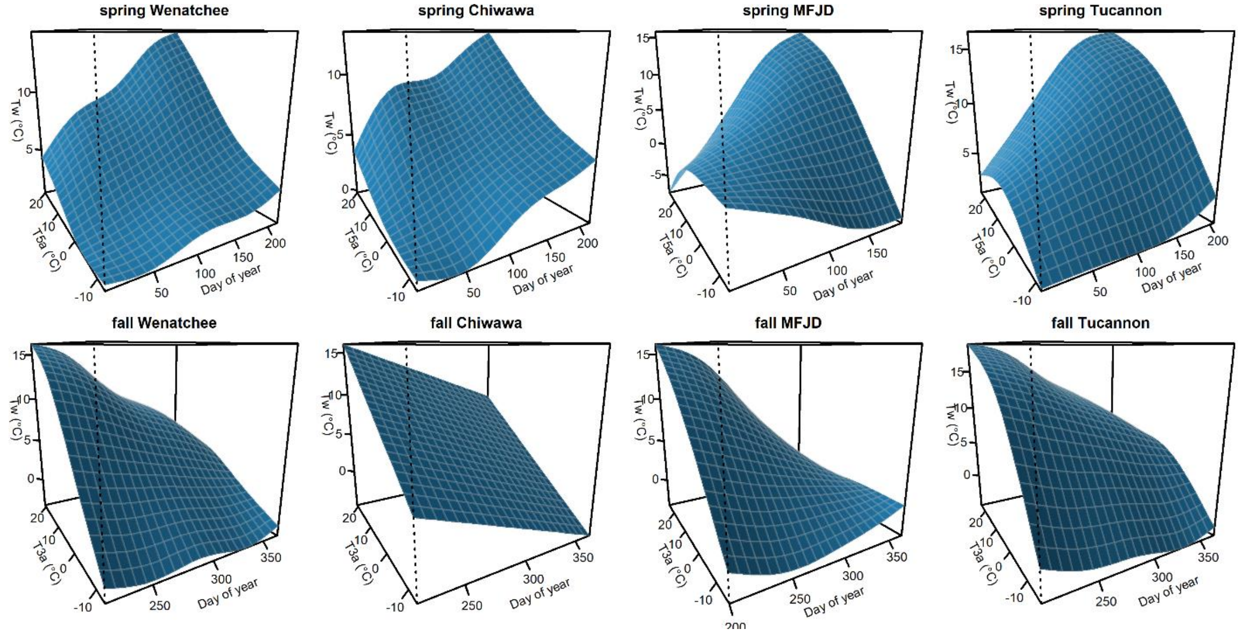

Figure SD2: Conditional model surfaces showing the interacting relationship between averaged air temperature variables (*$T5_a$* or *$T3_a$*) and *Day of year* (*D*) for the best spring and fall models in all study basins

Flow **(*F*) interacting with averaged air temperature variables (*$T5_a$* or *$T3_a$*)**

Flow affects the sensitivity of streams to meteorological conditions by altering thermal inertia and the residence time of water. Air temperature variables interacting with *Flow* represented one of the most influential effects in these models. In alignment with hypothesized effects, we found that discharge has a strong mitigating effect on stream temperatures during the spring warming period, reducing stream temperatures when air temperatures are hot and increasing stream temperatures when air temperatures are cold. *Flow* during the spring warming period is strongly related to the magnitude of snowpack melt, which also leads to depressed stream temperatures when air temperatures are high. Consequently, *Flow* in the spring appears to not only account for the above described effects of increased thermal inertia and reduced residence times of water, but also the effect of snowmelt cooling, as evidenced by the larger depression in stream temperature at higher discharges in spring models compared to fall models. Accordingly, we found no need to include the effect of *Snow Depth* in spring models but did include it in fall models (Figure A5).

Surprisingly, we found that this interaction was not useful in the fall warming period models for the M.F. John Day and the Tucannon. We conjecture that this may be a consequence of the high influence of cool groundwater springs in these basins which become a proportionally larger contributor to total flows at lower discharges. Parts of these basins see limited temperature variability during the late summer/early fall low flow period which would be consistent with a large groundwater signal. Identified groundwater sources could be included in future models.

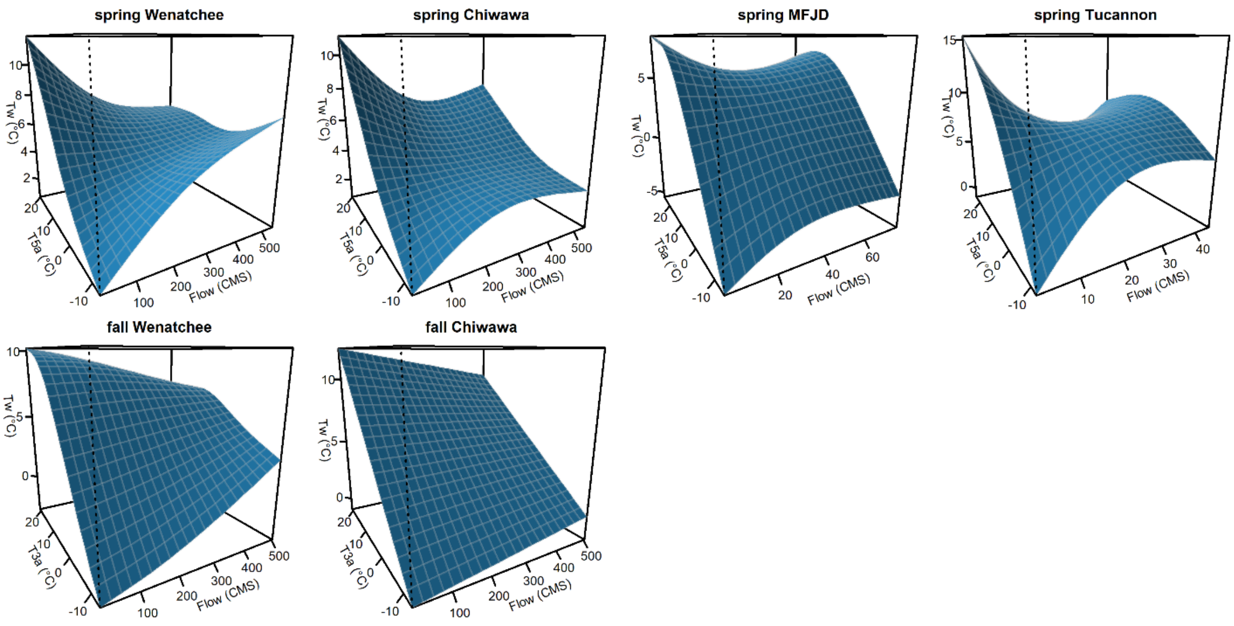

Figure SD3: Conditional model surfaces showing the interacting relationship between averaged air temperature variables (*$T5_a$* or *$T3_a$*) and *Flow* (*F*) for the best spring and fall models in all study basins.

Snow April 1st (*SA1*) depth interacting with *Day of year* (*D*)

An interaction between *Snow April 1st* and *Day of year* in the spring warming period models was consistently useful in describing a cooling effect of larger annual snowpacks on late-spring and summer stream temperatures. Note, *Snow April 1st* is included as a single annual value in the model, and not as a temporally continuous variable. The interaction suggests that *Snow April 1st* has a minimal influence on stream temperature early in the spring but a larger influence late in the period when air temperatures are warm. In the Wenatchee and Chiwawa basins, which are heavily influenced by snowpack, the effect of Snow April 1st was found to extend into the fall warming period. While the effect of daily values of snowpack is largely captured by *Flow* in the spring models as described above (Figure A4), rain and groundwater sources are likely to compose a larger proportion of total flows later in the spring period as snowpack diminishes. These water sources are likely to be warmer than snowpack melt. However, during high snowpack years the influence of snowpack is likely to persist longer, and thus stream temperatures are likely to remain cooler later into the spring warming period and the beginning of the fall cooling period.

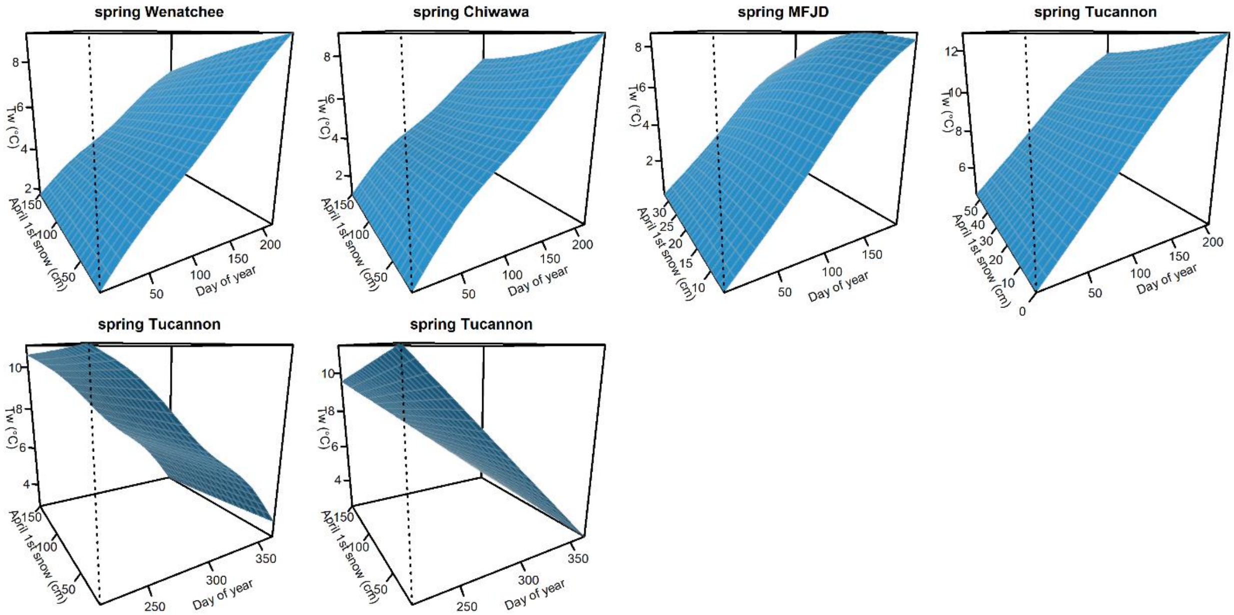

Figure SD4: Conditional model surfaces showing the interacting relationship between *Day of year* (*D*) and *Snow April 1st* (*SA1*) for the best spring and fall models in all study basins.

***Snow Depth* (*S*) interacting with averaged air temperature ($T3_a$)**

While the effect of daily values of *Snow Depth* was largely captured by *Flow* in spring warming season models, fall models were improved by the inclusion of this variable interacting with averaged $T3_a$. As with *Flow*, models suggested that when snowpack is present, it mitigates the effects of high air temperatures on stream temperatures. Including this relationship may account for the difference between the strength of the spring and fall relationships between air temperature variables and *Flow* (Figure A3). The models also suggested a positive relationship between stream temperature and *Snow Depth* at air temperatures below freezing. This may be a consequence of snowpack insulating streams from air temperatures, though the exact nature and the consistency of this relationship requires further exploration.

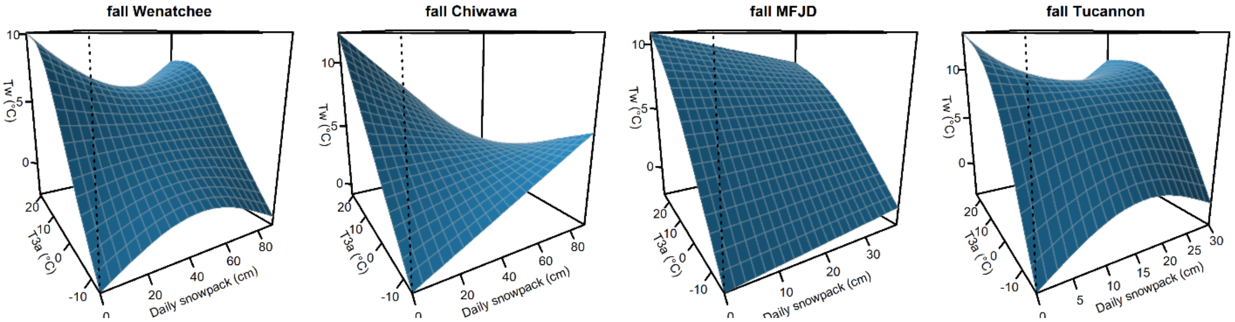

Figure SD5: Conditional model surfaces showing the interacting relationship between daily values of daily *Snow Depth* (*S*) and averaged air temperature ($T3_a$)for the best fall models in all study basins.

SPATIAL x CLIMATE RELATIONSHIPS AND INTERACTIONS

***Catchment elevation* (*E*) interacting with *Day of year* (*D*)**

High elevation mountainous areas contribute cooler waters to downstream sites as a consequence of lower local air temperatures. Since our measure of air temperature was not spatially explicit, including information on elevation accounts for lapse rates in air temperature within a watershed. Additionally, mountainous headwaters often contain a proportionally higher snowpack and groundwater influence which leads to cooler temperatures during the spring-melt and summer low-flow period. The effect of *Catchment elevation* changes seasonally in the study watersheds; sites with higher elevation catchments have substantially cooler stream temperatures during warm summer months, however the effect of *Catchment elevation* appears to be minimal in the winter during the beginning of the spring warming period. As previously discussed, this is likely a consequence of minimal spatial and interannual variability in stream temperature in the study watersheds during the winter period when air temperatures tend to be near or below zero, even at low elevations.

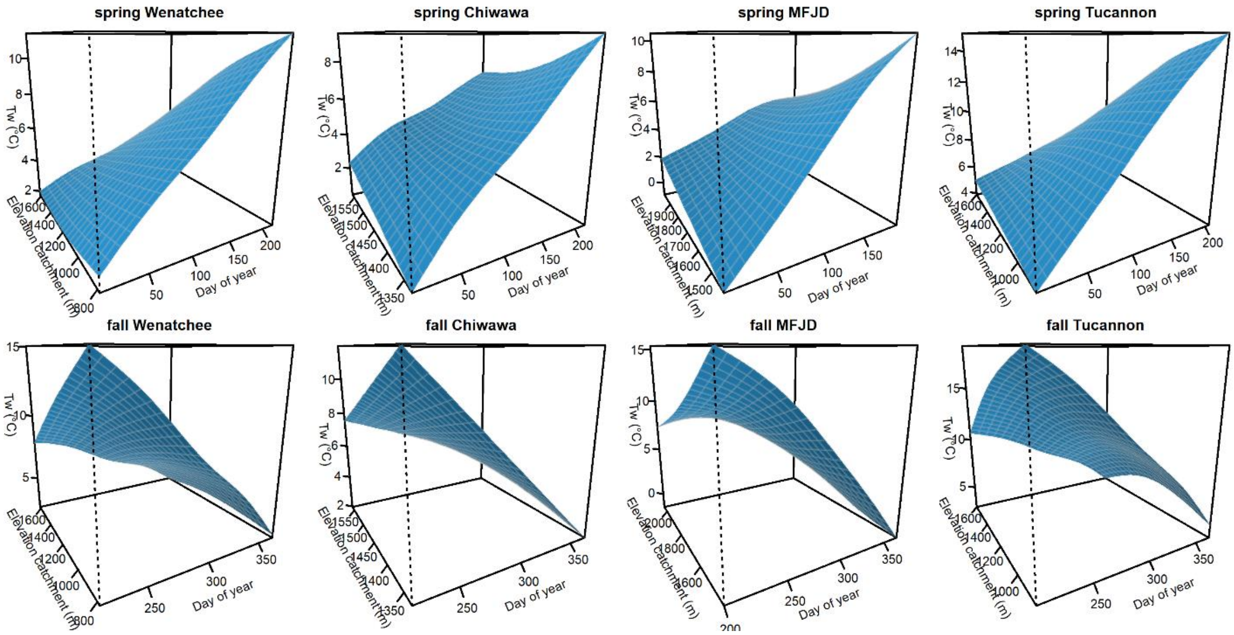

Figure SD6: Conditional model surfaces showing the interacting relationship between the *Catchment elevation* (*E*) and *Day of year* (*D*) for the best spring and fall models in all study basins.

***Elevation change (EΔ)* interacting with averaged air temperature variables (*$T5_a$* or *$T3_a$*)**

Due to correlation between the site-specific elevation and *Catchment elevation* (*E*), we utilized *Elevation change* (*EΔ*), which represents the difference between *Catchment elevation* and the site-specific elevation of the predicted reach and used to limit collinearity. Thus, *Elevation change* represents an estimate of the average change in elevation from a streams source to the logger site and is always negative. *Elevation change* interacting with averaged air temperature variables ($T5_a$ or $T3_a$) was utilized in all models for both seasons. In general, results suggests that *Elevation change* has a stong effect in some basins high air temperatures, but generally a minimal effect at air temperatures near freezing. At high air temperatures, sites with bigger negative value of *Elevation change,* and thus located at lower elevations, are likely to have higher stream temperatures than those located at elevations near headwaters. Conversely, sites at elevations nearer to headwaters sources of snowmelt and groundwater are likely to be relatively cooler. Additionally, this effect also helps account for the effect of site-specific air temperatures by representing lapse rates with elevation. The effect of *Elevation change* was strongest in the Wenatchee River model (Figure A7), likely due to the most dramatic topographic differences between the headwaters and lower mainstems within the watershed. The variable was found to have a weaker effect in the Chiwawa and M.F. John Day.

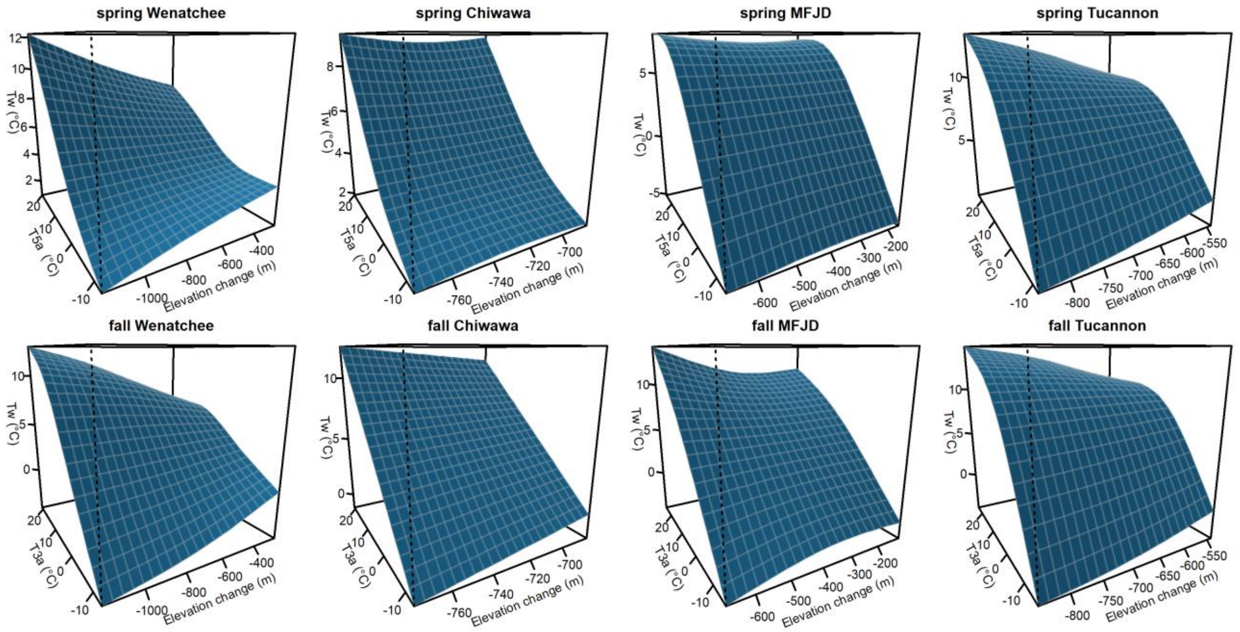

Figure SD7: Conditional model surfaces showing the interacting relationship between *Elevation change* (*EΔ*) and averaged air temperature variables ($T5_a$ or $T3_a$) for the best spring and fall models in all study basins.

Catchment elevation **(*E*) interacting with** ***Catchment area*** **(*A*)**

The modeled relationship between *Catchment elevation* and *Catchment area* suggests that the coolest temperatures in watersheds are generally found in high elevation headwaters, while the warmest temperatures are found at low elevation mainstems. Water at sites with larger catchment areas has likely been exposed to atmospheric conditions for a longer time relative to headwater sites. Thus, these sites tend to have higher stream temperatures during the summer warm weather season. In the MF John Day and the fall Chiwawa models the interaction suggests that *Catchment elevation* is more influential at sites with higher *Catchment areas*. While this interaction was found to be important for prediction accuracy, interpreting distinctions between the watersheds requires further exploration. With the exception of the Chiwawa, general shapes of this interaction were largely consistent between seasons within watersheds.

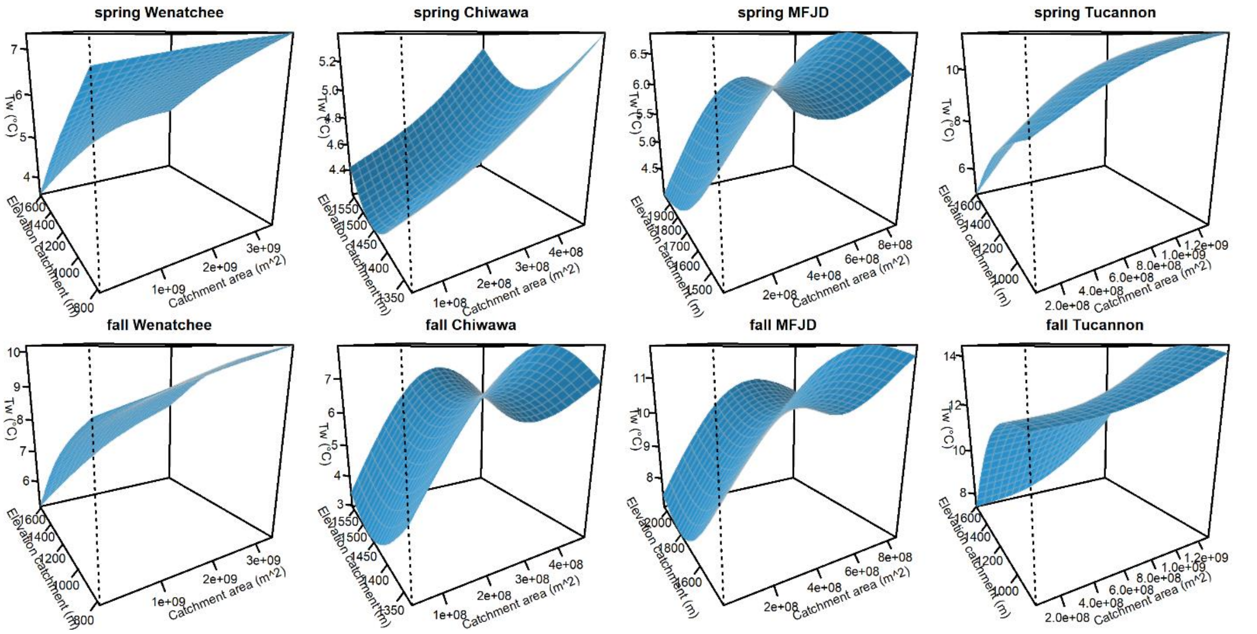

Figure SD8: Conditional model surfaces showing the interacting relationship between the *Catchment elevation* (*E*) and *Catchment area* (*A*) for the best spring and fall models in all study basins.

LOCAL RELATIONSHIPS

Local relationships were included in some models for some watersheds, but were not deemed to improve model fits or align with hypothesized effects in other models for other watersheds. As local relationships were less consistent in their importance and form, there is more uncertainty in the interpretation of the mechanisms that they are capturing in comparison to relationships deemed universal.

Catchment Area **(*A*) interacting with averaged air temperature variables (*$T5_a$* or *$T3_a$*)**

The effect of *Catchment area* interacting with averaged air temperature variables ($T5_a$ or $T3_a$) was retained in all models for the Wenatchee and the Tucannon and fall period models for the M.F. John Day. In these models the warming effect of *Catchment area* was negated at air temperatures near and below freezing. This is likely a consequence of low temperature variability across the basin during periods with near or below freezing air temperatures. While this interaction was not particularly strong in most of the study basins, it improved fits and predictions and thus was incorporated in most models.

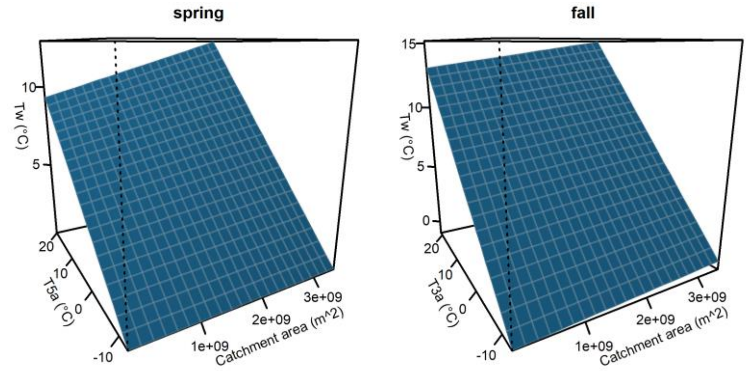

Figure SD9: Conditional model surfaces of *Catchment area* (*A*) interacting with averaged air temperature variables ($T5_a$ or $T3_a$) for the linear Wenatchee models.

Catchment Area **(*A*) interacting with *Flow* (*F*)**

Catchment Area interacting with *Flow* improved the fit and validation testing dataset predictions for most watersheds. For the Wenatchee, the effect of discharge is reduced and near zero at low catchment areas. Headwater sites are likely proportionally more affected by nearby groundwater sources and snow melt and thus may not be as sensitive to changes in environmental conditions described by *Flow* than stream sites with larger catchment areas. However, this relationship may be a consequence of utilizing a single measure of discharge from a gage on the mainstem. Consequently, the *Flow* metric may not be informing conditions at small headwater sites well. The exact source and the consistency of this interaction should be further investigated.

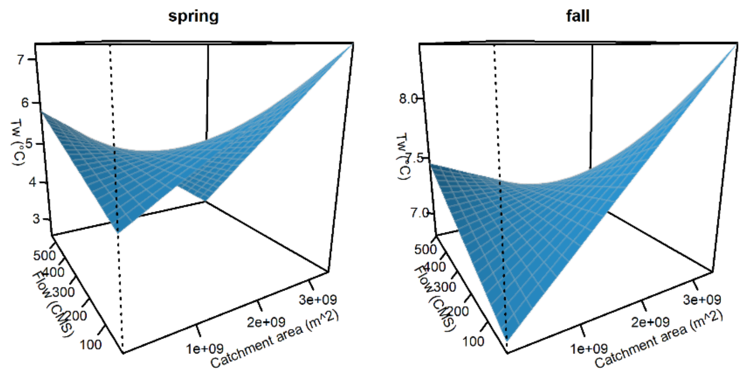

Figure SD10: Conditional model surfaces of *Catchment Area* (*A*) interacting with Flow *(F)* on stream temperature for the Wenatchee GAM models.

***Catchment elevation* (*E*) interacting with *Snow Depth* (*S*)**

The interaction between *Catchment elevation* and *Snow Depth* was retained in all fall models and had the clearest effect in the Chiwawa and the M.F. John Day. The interaction suggest that *Snow depth* has a slightly larger cooling effect on stream temperatures at higher elevations. The snowpack variable came from a single point source at high elevation in each watershed and was not spatially continuous. Higher elevation catchments tend to accrue more snow in comparison to lower elevation catchments and thus are likely to be more affected by snow melt. However, the strength of this interaction tended to be relatively minor and somewhat inconsistent.

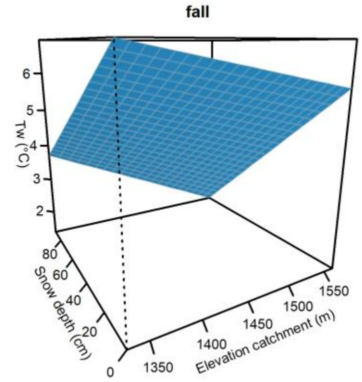

Figure SD11: Conditional model surface of *Catchment elevation* (*E*) interacting with *Snow Depth* (*S*) from the selected Chiwawa fall cooling season linear model.

Base Flow Index (*BFI*) interacting with averaged air temperature variables (*$T5_a$* or *$T3_a$*)

BFI represents the estimated mean low flow divided by the mean annual discharge and is available for all stream reaches of the study watersheds within the National Hydrography Dataset. Streams with higher *BFI* are likely more influenced by groundwater in comparison to streams with low *BFI*. We expected high *BFIs* to reduce stream temperatures at high air temperatures during the low-flow summer period. While we saw this effect in some of the Wenatchee and M.F. John Day models, we did not find this effect consistently across all models and the effect was fairly small when retained. Other regional modeling efforts have found this variable more useful. It may be that within the scale of our watersheds this variable is not precise enough to be very informative but on larger spatial scales would be more useful.

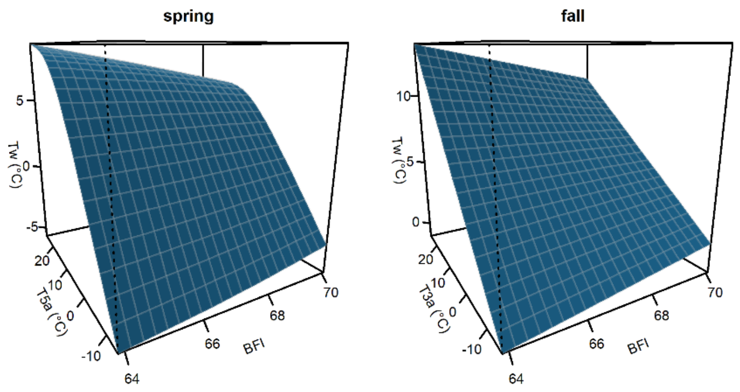

Figure SD12: Conditional model surfaces of *Base Flow Index* (*BFI*) interacting with air temperature variables ($T5_a$ or $T3_a$) for the M.F. John Day spring warming season GAM and the Wenatchee fall cooling season linear models.

Forest Cover (catch) **interacting with air temperature variables (*T_a AVG 3/5*)**

We considered forest cover for the entire catchment (*FC*) and more locally by reach contributing areas (*FR*). We expected forest cover to produce a stream temperature mitigating effect through providing shading and retaining moisture within the system. Forest cover in either form did not have a consistent relationship with stream temperature across models. *FC* was utilized in the M.F. John Day models while *FR* was only utilized in the spring Wenatchee models. While the effect of stream shading has consistently been shown to be important in other modeling studies, there may be a couple of reasons why we didn't see consistent effects of the forest cover variables in our analysis. First, the Wenatchee, Chiwawa, and to a lesser extent, the M.F. John Day contain alpine areas at high elevations which have low percent forest covers. Since high elevation areas also produced streams with cool temperatures due to the influence of snow melt and groundwater, this could have could have obscured the effect of the forest cover variables at lower elevations. Additionally, while *FR* was an attempt to account for reach specific shading by riparian canopy, it likely does a poor job of this as the RCA units were much larger than the riparian zone that is likely to determine stream shading. Accordingly, a variable that provides more accurate estimates of riparian shading would likely improve models.

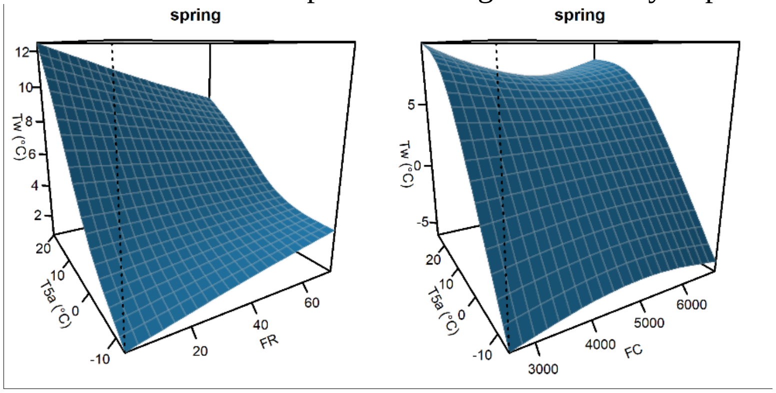

Figure SD13: Conditional model surface of reach summarized forest cover (*FR*) interacting with air temperature ($T5_a$) for the Wenatchee spring GAM model and catchment summarized forest cover (*FC*) interacting with air temperature ($T3_a$) for the M.F. John Day fall GAM model.

Slope (SL) interacting with averaged air temperature variables ($T5_a$ or $T3_a$)

Water moves faster in watersheds with higher slopes and thus has shorter residence times. Accordingly, we would expect *Slope* to mitigate changes in stream temperature caused by high air temperature. This interaction was retained in 9 out of 16 models, though in none of the models for the Tucannon. As the Tucannon was fit to a linear network representing the mainstem, this model only required spatial information from the elevation and catchment area variables. Information on stream slope would inherently be captured by these variables in a linear network. Some of the information provided by *Slope* may have been retained by including both *Catchment elevation* (*E*) and *Elevation change* (*EΔ*) variables, which may have precluded its usefulness in some of the other models by describing some of the same information.

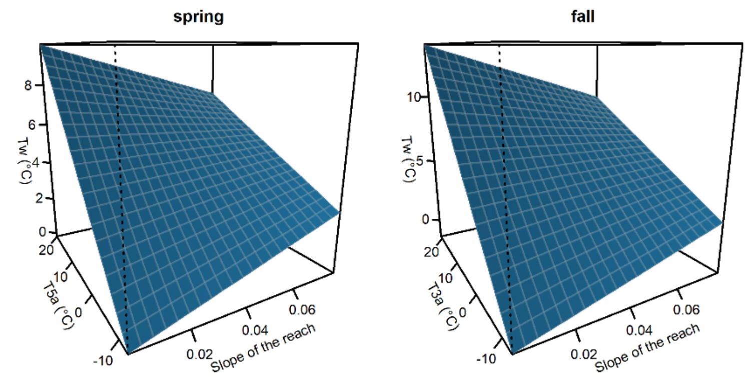

Figure SD14: Conditional model surfaces of reach *Slope* (*SL*) interacting with air temperature ($T5_a$ or $T3_a$) for the selected Wenatchee linear models.

***Lakes* (*L*) interacting with *Day of year* (*D*)**

The Wenatchee was the only study basin with a substantial presence of lakes, with Lake Wenatchee (10 km^2) affecting the water temperatures of mainstem sites downstream of the lake. Lakes increase the residence time of water within watersheds; surface waters that warm in lakes during the summer usually raise temperatures of downstream sites. In both GAM and linear regression models, an interaction with *Day of year* improved model RMSE over an interaction with air temperature variables. Due to the large water volume of lakes, they are likely to be less affected by fluctuating daily air temperatures leading to a smoother seasonal effect better represented by an interaction with *Day of year*. While we only saw a strong effect of this variable in the Wenatchee model, it has been described in other investigations.

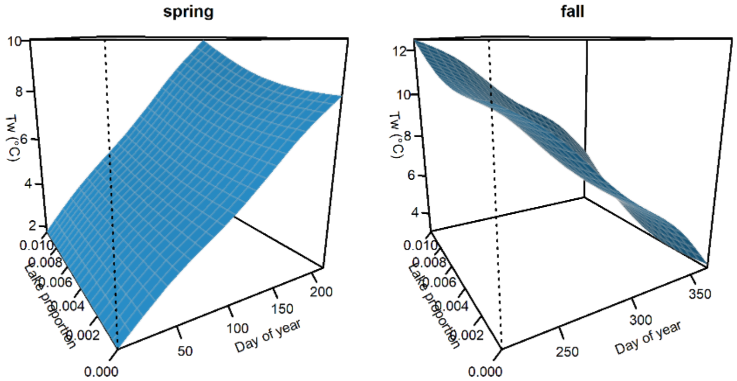

Figure SD15: Conditional model surfaces for the *Lakes* (*L*) interacting with *Day of year* (*D*) from the selected Wenatchee GAM models.

Glaciers

We did not find an effect of *Glaciers* in GAM or linear regression models. Glaciers only existed in the Chiwawa and Wenatchee watersheds. However, the small size of the glaciers in these watersheds may have had too small of an effect to be captured. Additionally, few loggers were located within close proximity to glaciers. Thus, the expected cooling effect of glaciers may have been largely attenuated by the time glacier melt reached loggers.
