# Supplementary figures and images for: Accurate spatiotemporal predictions of daily stream temperature from statistical models accounting for interactions between climate and landscape
Source: PeerJ. 2019 Nov 12;7:e7892. doi: 10.7717/peerj.7892 (PMC6857678; doi:10.7717/peerj.7892)

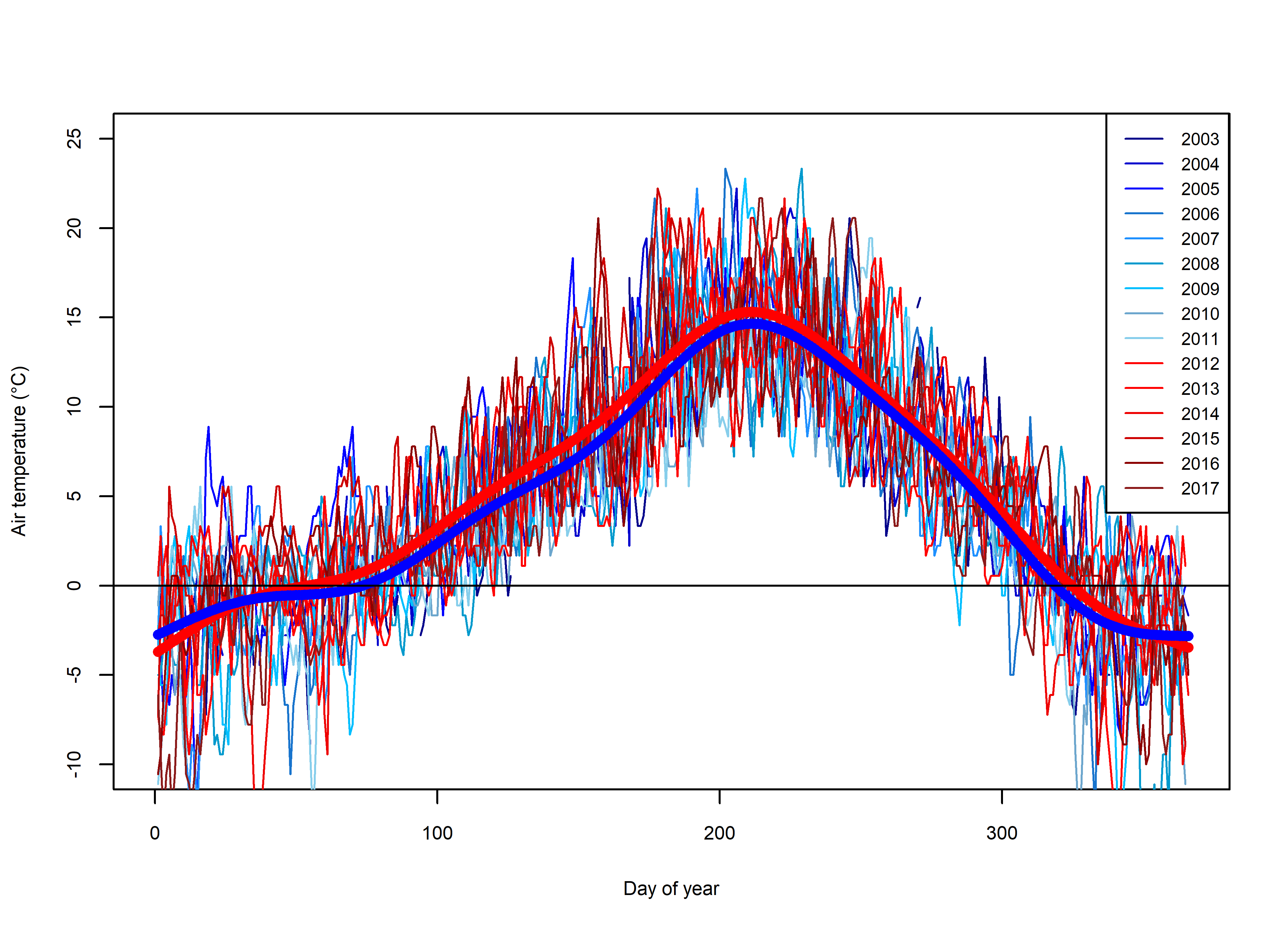

Supplement: Figure S1 — Daily mean air temperature measurements for the Wenatchee for all years in the fitting dataset (red) and validation dataset (blue). Smoothed GAM fit for all years of the fitting and validations datasets are shown by thick red and blue lines respectively. [file peerj-07-7892-s002.png]

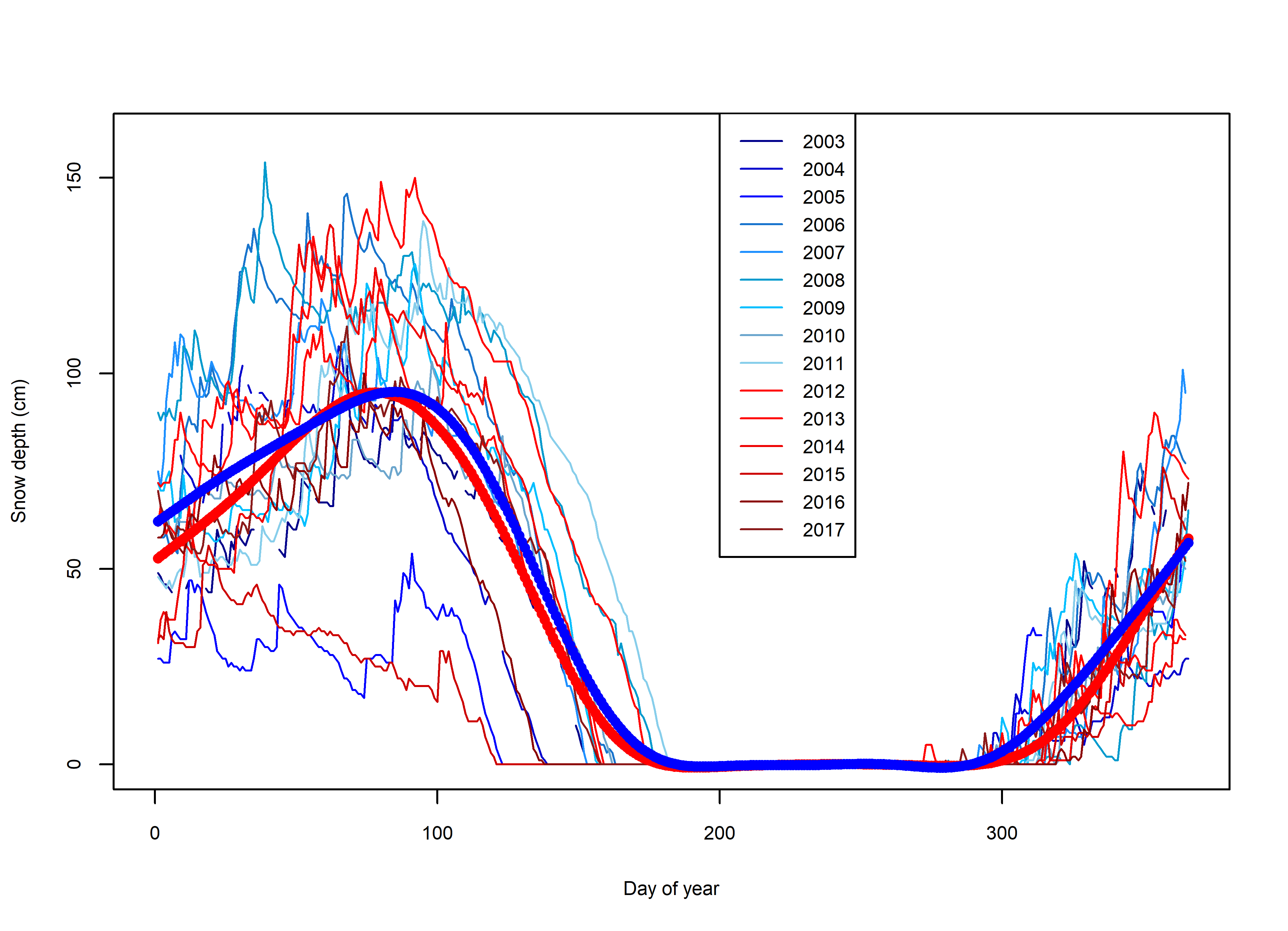

Supplement: Figure S2 — Daily mean snow depth measurements for the Wenatchee for all years in the fitting dataset (red) and validation dataset (blue). Smoothed GAM fit for all years of the fitting and validations datasets are shown by thick red and blue lines respectively. [file peerj-07-7892-s003.png]

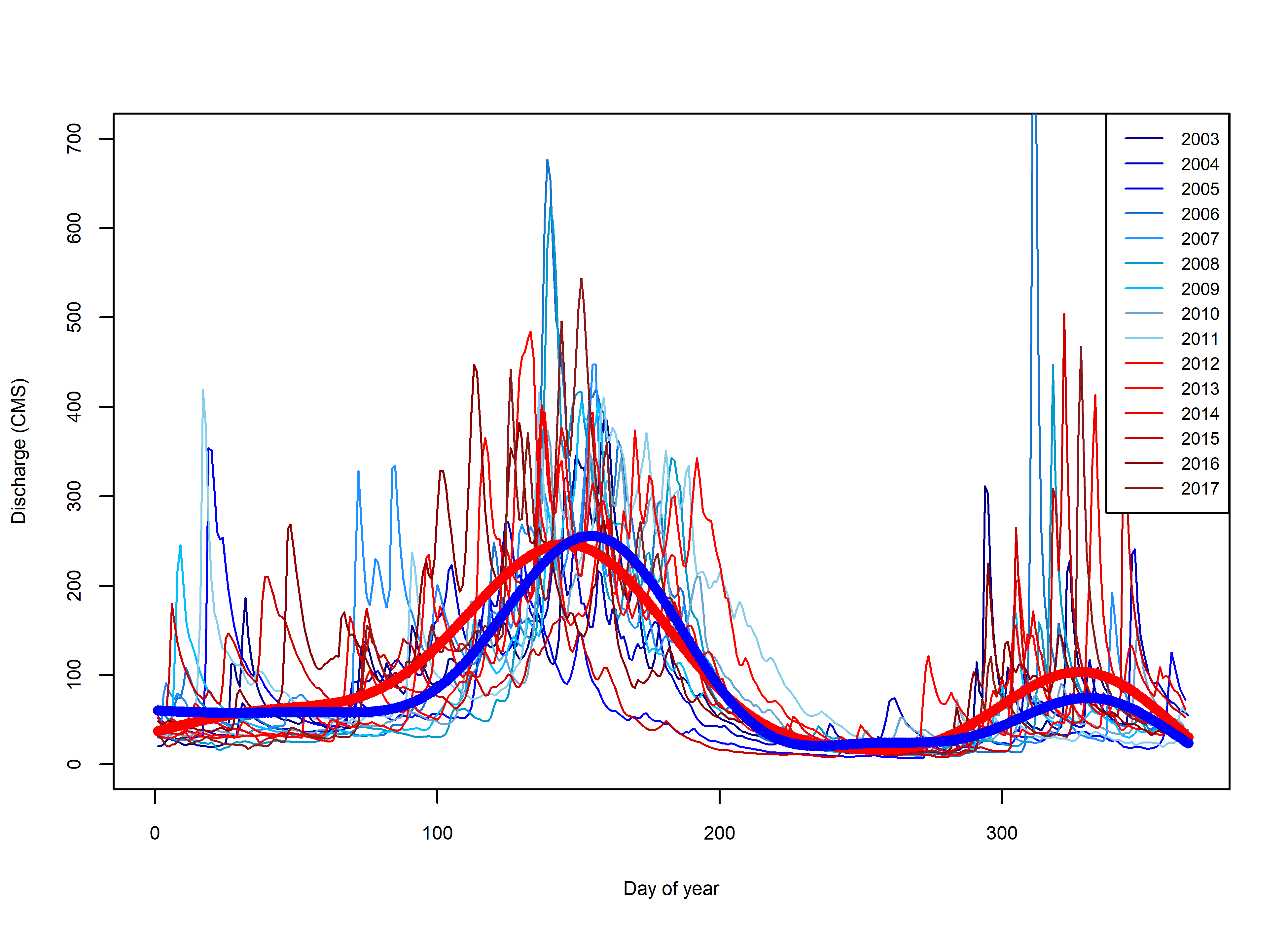

Supplement: Figure S3 — Daily mean discharge measurements for the Wenatchee for all years in the fitting dataset (red) and validation dataset (blue). Smoothed GAM fit for all years of the fitting and validations datasets are shown by thick red and blue lines respectively. [file peerj-07-7892-s004.png]

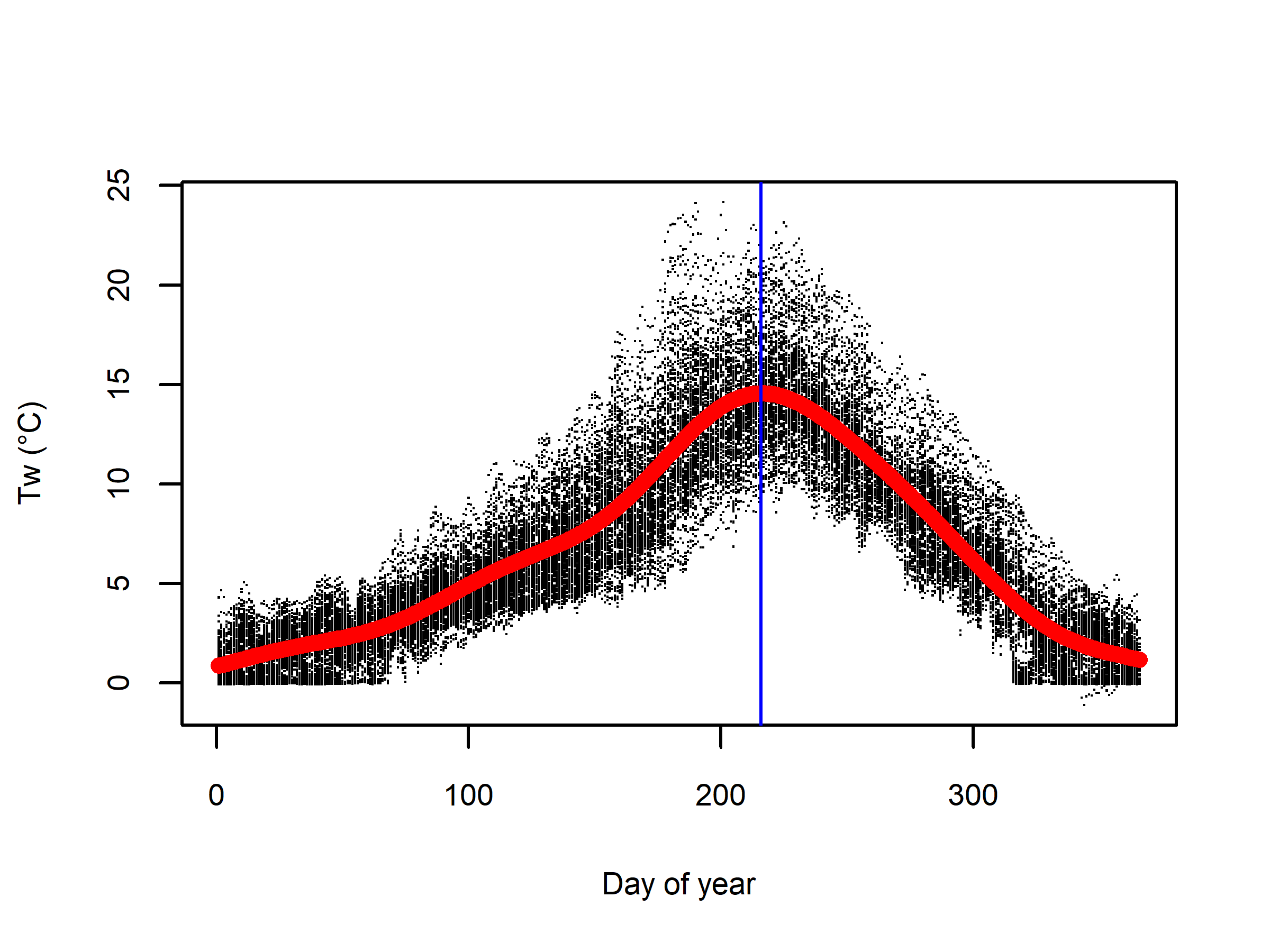

Supplement: Figure S4 — All stream temperature in the Wenatchee fitting dataset shown by day of year with GAM smoother shown by red line and date of predicted max temperatures marked by blue line. Dates of predicted max temperature in fitting datasets were utilized to split year into spring warming and fall cooling periods for model fitting and subsequent validation predictions. [file peerj-07-7892-s005.png]

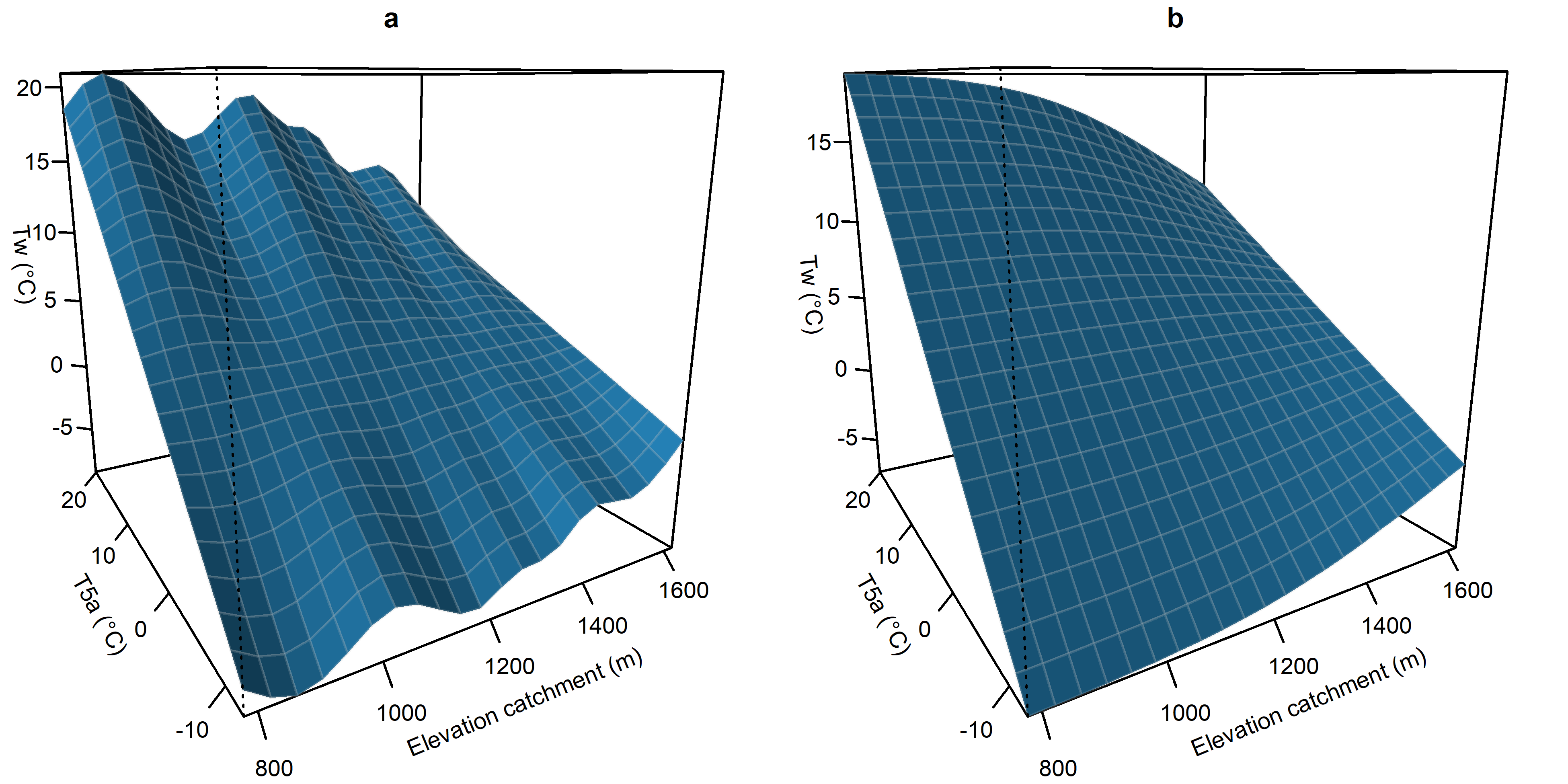

Supplement: Figure S5 — Example of GAM models fit utilizing Wenatchee River fitting dataset data with effects of averaged air temperature the five days before the predicted day (T5_a) and the average catchment area elevation (AE) utilizing penalized regression splines to determine the number of knots (a) and with knots specified at 3 (b). Using penalized regression splines produces a “crumpled blanket effect”, which is overfit and does not align consistently with hypothesized effects of the variables. [file peerj-07-7892-s006.png]

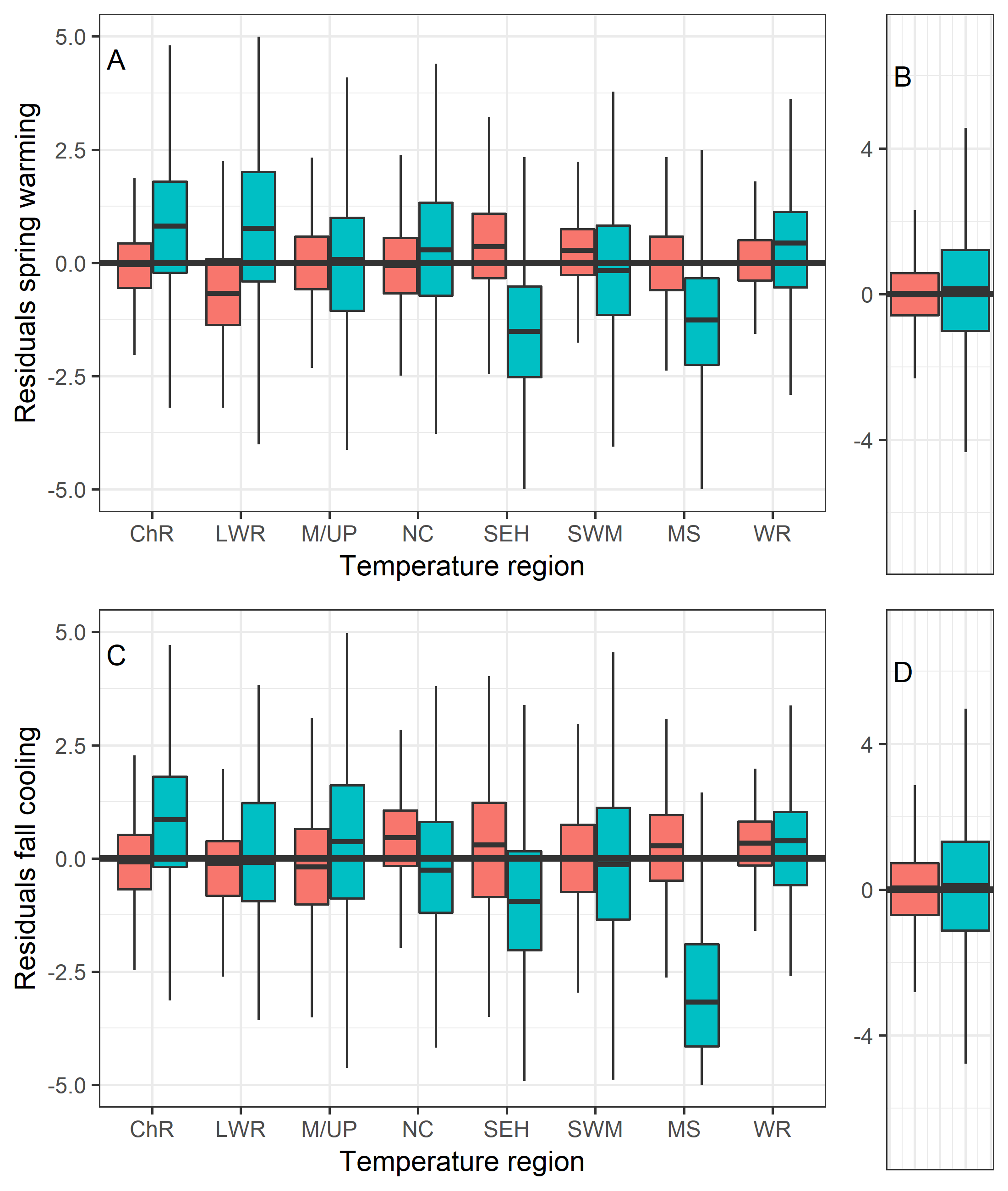

Supplement: Figure S7 — Boxplots showing the residuals by spatial regions (A and C) for the Wenatchee selected GAM spring (A) and fall (C) models, as well as for all residuals combined (B and D) in red compared against analogous predictions from a smoother for day of year fit to the entire basin in turquoise (smoother shown in Fig. S4). Selected models reduce spatial biases. Spatial regions analyzed are shown in Fig. S6 (ChR is Chiwawa River, LWR is Little Wenatchee River, M/UP is Mission and Upper Peshastin Creeks, NC is Nason Creek, SEH is Southeast Hills, SWM is South West Mountains, MS is Mainstem, WR is White River) [file peerj-07-7892-s008.png]
