# Supplementary material for: Accurate spatiotemporal predictions of daily stream temperature from statistical models accounting for interactions between climate and landscape
Source: PeerJ. 2019 Nov 12;7:e7892. doi: 10.7717/peerj.7892 (PMC6857678; doi:10.7717/peerj.7892)

Wenatchee temperature regions

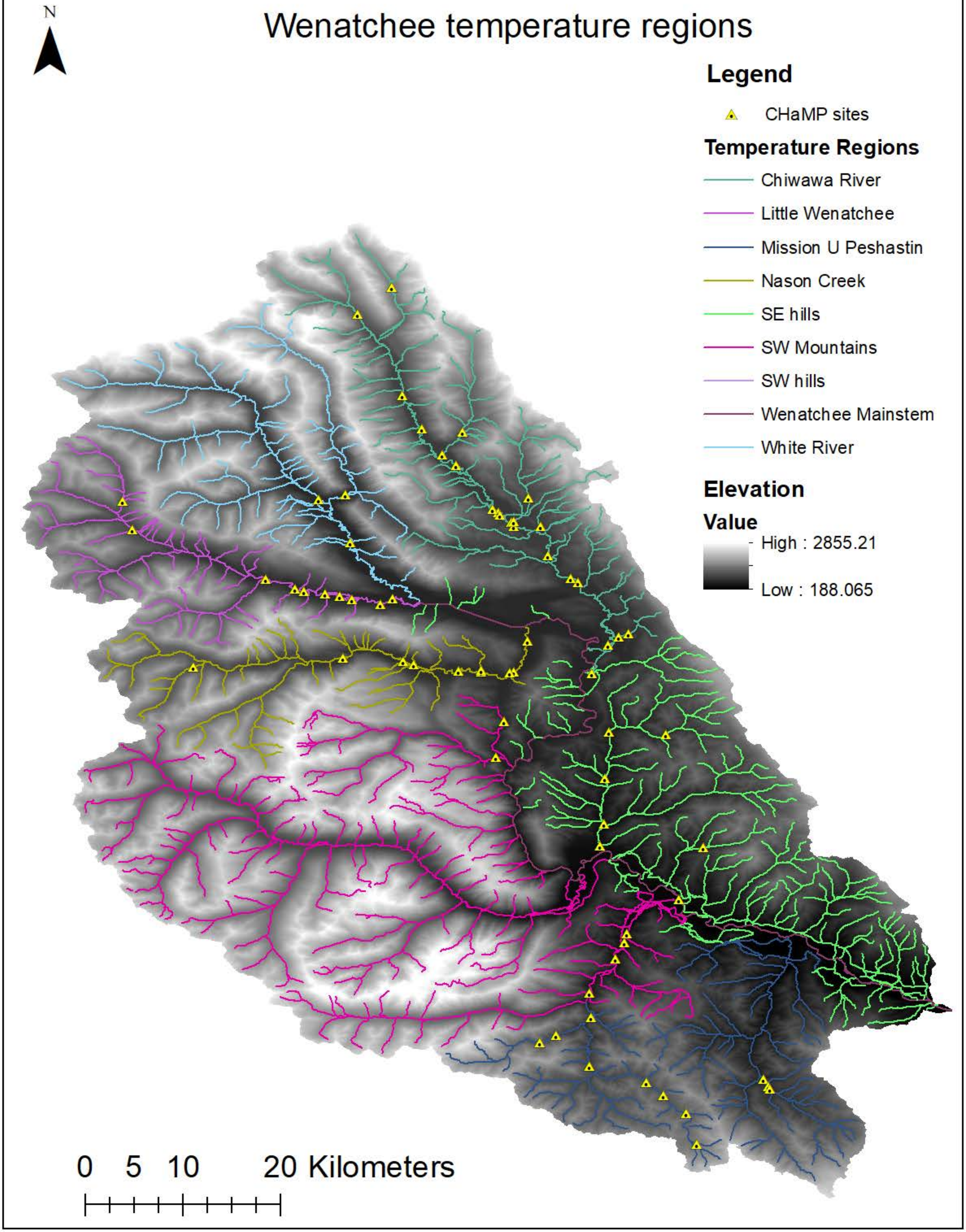

Supplement: Figure S6 — Model training dataset prediction biases from selected GAM models are shown for these spatial regions in Fig. S7. [file peerj-07-7892-s007.pdf]
